# Rootstock effect on horticultural performance and fruit quality is not uniform across five commercial apple cultivars in western New York

**DOI:** 10.3389/fpls.2025.1552625

**Published:** 2025-03-10

**Authors:** Brian T. Lawrence, Gennaro Fazio, Luis Gonzalez Nieto, Terence L. Robinson

**Affiliations:** ^1^ School of Integrative Plant Sciences, Horticulture Section, New York State Agricultural Experiment Station, Cornell University, Geneva, NY, United States; ^2^ U.S. Department of Agriculture, Agricultural Research Service (USDA ARS) Plant Genetic Resources Unit, Cornell AgriTech, Geneva, NY, United States; ^3^ Fruit Production Programme, Institute of Agrifood Research and Technology (IRTA), Lleida, Catalonia, Spain

**Keywords:** cultivar-rootstock interaction, orchard production, yield, fruit size, fruit firmness, fruit red color

## Abstract

**Introduction:**

The interactive effect of different apple scions with commonly used rootstocks could result in growers selecting an inferior option for tree survival, yield, and fruit quality.

**Methods:**

The long-term tree performance and fruit quality interactions of 19 rootstocks (including Budagovsky, Geneva, and Malling series) and 5 apple cultivars (‘Empire’, ‘Gala’, ‘Honeycrisp’, ‘Mustu’, and ‘Delicious’) were explored in two orchards in Western New York. The first orchard examined the five cultivars on dwarfing rootstocks (B.9, CG.4210, G.11, G.16, G.202, G.41, G.65, G.814, M.26, M.9Pajam2, and M.9T337) and was planted at a spacing of 1.22 m x 3.66 m (2,243 trees ha^-1^). The second orchard examined the same cultivars on semi-dwarfing rootstocks (B.118, G.214, G.30, G.210, G.935, G.222, M.26, and M.7) and was planted at a spacing of 1.83 m x 4.27 m (1,282 trees ha^-1^).

**Results:**

Following 17 years, the variables of tree mortality, growth, cumulative yield, and cumulative yield efficiency each resulted in a significant interaction between cultivar and rootstock in both orchards. There were no significant interactions on quality variables measured except fruit color of the 3 bi-colored ‘Gala’, ‘Honeycrisp’ and ‘Delicious’ for both the dwarfing and semi-dwarfing rootstocks.

**Discussion/Conclusions:**

The implications of the interactions observed are that apple producers should pair specific rootstocks with specific cultivars to optimize orchard performance.

## 1 Introduction

Planting and maintaining orchards is an inherently long-term investment for growers and multiple decisions such as tree spacing, training system, cultivar, and rootstocks largely determine horticultural success and economic profitability (Autio et al., 2017; Gonzalez Nieto et al., 2023; Reig et al., 2020; Robinson et al., 2007). Within the past century, the appearance and efficiency of orchards have dramatically changed as the use of dwarfing rootstocks enabled closer tree spacing and training systems, such as the tall spindle, have become commonplace. Understanding which rootstocks perform best over time when paired with different scions enables growers to maximize profitability. Particular rootstock genotypes are thought to convey a similar effect to tree growth habit and performance regardless of the scion (Marini and Fazio, 2018; Tworkoski and Miller, 2007). However, rootstock traits, such as dwarfing, are not always conveyed similarly to different scion cultivars (Fazio, 2021) and rootstock-scion interactions have been previously identified regarding plant habit (Tworkoski and Miller, 2007) and horticultural performance (Autio et al., 2001). The long-term understanding of such interactions on horticultural performance is largely missing in the literature, and exploration of scion-rootstock interactions may allow growers to establish desired cultivars paired to what have been coined “designer rootstocks”, optimizing the horticultural and economic potential of an orchard (Fazio and Robinson, 2021).

While new apple cultivars are frequently released for superior eating and storage qualities, rootstock generation has been historically slower. Nonetheless, several breeding programs have released new rootstock genotypes, each selected to withstand the specific challenges of their region of the world. The oldest of the rootstock options still used in modern production are from the Malling series (M or MM) from Kent, England, and are often used as the vigor standards in comparative rootstock research experiments (Autio et al., 2017). Winter hardy introductions by I.V. Budagovsky (Bud or B) from the Tambov region of Russia have also been previously explored. The Cornell-Geneva breeding program (CG or G) in New York, USA, also has released many rootstocks which have disease resistance, especially against fire blight (*Erwinia amylovora*), woolly apple aphid (*Eriosoma lanigerum)* and replant disease (Fazio et al., 2013). All rootstocks are generally classified by relative size to a standard, such as an apple seedling, and their ability to restrict the growth of the attached scion. If left unpruned, dwarfing stocks generally restrict tree size by 50% or more while semi-dwarfing stocks restrict tree size by 25-50%. In recent decades, commercial growers have preferred more dwarfing rootstocks which allow for closer tree planting and can greatly improve orchard profitability (Lordan et al., 2019; Reig et al., 2019b).

Due to the varying return on investment differences between establishment decisions such as orchard spacing (Ho et al., 2024), as well as rootstock and scion combinations (Lordan et al., 2019; Gonzalez Nieto et al., 2023), the objective of this work was to identify possible long-term (17 year) horticultural interactions between cultivar and rootstock at two different orchard spacings. Additionally, the interactions to fruit quality variables were also explored from the final 4 years of the orchard lifetime. We hypothesized that the rootstock influence would not be consistent across the tested cultivars and a significant interaction would be present between cultivar and rootstock for several horticultural (tree survival, tree size, cumulative yield, cumulative yield efficiency) and fruit quality measurements (fruit size, firmness, soluble solids and color).

## 2 Materials and methods

### 2.1 Experimental location, plant material, and design

Two orchards, both planted in 2007 at Cornell’s AgriTech Campus in Geneva, New York, USA (42°51′ N, 77°01′ W) were used for this study. The orchards were planted at the same location side-by-side, on soil classified as Lima loam, which has a high water-holding capacity and moderate drainage (USDA). Both orchards were irrigated by drip during the growing season, while pruning and chemical control treatments for weeds and pests followed industry standards.

The bi-color cultivars ‘Empire’, ‘Gala’ and ‘Honeycrisp’, the green cultivar ‘Mutsu’, and the fully red cultivar ‘Delicious-Super Chief strain’, were evenly divided between 18 rootstocks designated as ‘Dwarfing’ (experiment 1) or ‘Semi-dwarfing’ (experiment 2). The rootstocks included two from the Budagovsky series (B.118 and B.9), twelve unreleased or released stocks from the Cornell-Geneva breeding program (CG.4210, G.11, G.16, G.202, G.210, G.214, G.222, G.30, G.41, G.65, G.814, and G.935) and four originating from the Malling series (M.26, M.7, M.9Pajam2, and M.9T337). A total of 110 trees of each cultivar (55 trees per single row) were paired with the ‘Dwarfing’ rootstocks (B.9, CG.4210, G.11, G.16, G.202, G.41, G.65, G.814, M.26, M.9Pajam2, M.9T337) for experiment 1. A total of 90 trees of each cultivar (45 trees per single row) were paired with ‘Semi-Dwarfing’ rootstocks (B.118, G.210, G.214, G.222, G.30, G.935, M.26, and M.7) for experiment 2. M.26 was included in both experiments as a comparative control.

Experiment 1 with dwarfing stocks was planted at a spacing of 1.22 m x 3.66 m (2,243 trees ha^-1^) and experiment 2 with the semi-dwarfing stocks was planted at a spacing of 1.83 m x 4.27 m (1,282 trees ha^-1^). The orchards were designed as split plots with the cultivars planted in single rows and replicated twice in both experiments, while rootstocks (plots) divided each row into groups of 5 trees. In total, each cultivar and rootstock combination had a total of 10 trees per experiment.

### 2.2 Horticultural and fruit quality measurements

Field measurements were collected and calculated the same for both experiments. Tree survival was measured as the percentage of trees remaining after the year of orchard establishment. Trunk circumference was measured 30 cm above the graft union following the 2023 growing season and used to calculate final trunk cross sectional area (TCSA). Fruit number per tree and yield were measured annually. Yield was the sum of the weight of fruit harvested and an estimated weight of dropped apples from each tree. Dropped fruit weight was calculated using the average single-fruit weight multiplied by the number of dropped fruit from each tree for a given year. Fruit size was calculated as average weight of each apple from the ratio of yield and fruit number per tree. The cumulative yield was measured as the total of annual yield from each tree between 2009 and 2023. The cumulative yield efficiency (CYE) was calculated as the cumulative yield divided by the final (2023) TCSA measurement. Crop load adjusted fruit size was calculated by using crop load as a covariate and calculating the fruit size independent of crop load.

Fruit quality measurements were performed the same for both experiments. ‘Honeycrisp’ fruit were measured during 2019, 2021-2023, and other cultivars were measured between 2020-2023. A 25-50 fruit sample was taken from each of the four 5-tree sections of each rootstock and variety. Therefore, all samples had a minimum of 4 values to average every year except for ‘Delicious x G.16’, and only 3 samples were used for ‘Gala x CG.4210’ in experiment 1. Fruit samples were sorted and evaluated for size, mass, and color using a computerized fruit grading machine (CombiSort, GREEFA Grading Machines, Langstraat 12, 4196 JB Tricht, Netherlands). A subsample of ten fruit from each sample were used to measure average length (longitudinal section) and diameter (width) to calculate a length diameter ratio (L/D), fruit firmness (lbs) using a pressure texture machine (Fruit Texture Analyzer, QA Supplies LLC, Norfolk, Virginia), and total soluble solids (°brix) using a digital refractometer (Agato PR-101, Tokyo, Japan).

### 2.3 Statistical analysis

The basic model of the study was divided by experimental orchard (dwarfing or semi-dwarfing) and all variables of interest were examined by the fixed effects of cultivar, rootstock, their interaction, with tree number (replicate) added as a random factor. A covariate of crop load was additionally used to compare fruit size. Apart from measuring tree survival, G.16 was removed from the dwarfing rootstock experiment and G.210 was removed from the semi-dwarfing rootstock experiment prior to making other horticultural parameter comparisons due to ≥ 90% mortality. Fruit quality data were analyzed in a similar fashion, but each 5-tree plot resulted in a single measurement instead of individual tree replicates and year was included as a random effect. Fruit length, diameter, firmness and soluble solids were compared between all 5 cultivars while fruit color and size grading were examined between the three bicolor apples. All data were screened for outliers by cultivar and subjected to analysis of variance (ANOVA) using the Proc MIXED procedure of SAS (SAS Institute Inc., Cary, NC, USA). Overall main effect least square means were separated using Tukey’s honest significant (HSD) test. When significant interactions occurred, rootstock effect within each cultivar was examined using pair-wise t-tests and a Bonferroni correction (Autio et al., 2001) using an adjusted critical value of *P* = 0.001 for the dwarfing experiment and *P* = 0.002 for the semi-dwarfing experiment. The conservative approach allowed estimation of rootstock differences within each cultivar, to help the reader assess cultivar and rootstock patterns according to predetermined designations (dwarfing vs semi-dwarfing) as well as size classification (TCSA). To help the reader visualize the interactions of rootstock and scion cultivar we have graphed the data by cultivar with the least vigorous cultivar on the left and the most vigorous cultivar on the right with continuous lines connecting the rootstock data between cultivars. This method of graphing although not technically correct since cultivar is not a continuous variable, allows the reader to readily see the source of the interactions.

## 3 Results

### 3.1 Dwarfing rootstock horticultural performance

The dwarfing rootstock experiment had a significant interaction (*F* = 3.6, *P* ≤ 0.001) between cultivar and rootstock regarding tree survival after 17 years (**Table 1**). The ‘Empire’ trees had ≥ 90% survival on B.9, G.16, G.202, G.41, and M.26 while on CG.4210, G.11 and M.9Pajam2 had ≤ 40% survival. The ‘Delicious’ trees had ≥ 90% survival on B.9, G.11, G.202, G.814, and M.9Pajam2 but no surviving trees on G.16. The ‘Honeycrisp’ trees had ≥ 90% survival on most stocks including CG.4210 (100%), except for G.65 (70%) and B.9 (60%). The ‘Gala’ trees had ≥ 90% survival on B.9, G.11, G.41, G.814, M.26, and M.9T337 but only 20% survival on CG.4210. The ‘Mutsu’ trees had ≥ 90% survival on B.9, G.11, G.202, G.41, G.65, G.814, and M.9T337 while CG.4210 and G.16 had ≤ 50% survival. Across all stocks, ‘Honeycrisp’ and ‘Mutsu’ had the highest survival, and ‘Empire’ had the least surviving trees. Across cultivars, B.9, G.202, G.41, G.814 and M.9T337 had the highest survival while CG.4210 and G.16 had the lowest survival.

**Table 1:** Average survival (%) by cultivar and rootstock after 17 years (2007-2023) of the dwarfing rootstocks in Geneva, NY^z^.

Rootstock	Empire	Delicious	Honeycrisp	Gala	Mutsu	Mean
B.9	90 a	90 a	60 a	100 a	100 a	88 a
G.65	70 ab	80 a	70 a	80 a	100 a	80 ab
CG.4210	40 ab	50 ab	100 a	20 b	50 b	52 c
M.9T337	70 ab	80 a	100 a	100 a	90 ab	88 a
G.202	100 a	100 a	90 a	70 ab	100 a	92 a
G.41	90 a	60 a	100 a	90 a	100 a	88 a
G.16	90 a	0 b	90 a	80 a	60 ab	64 bc
G.11	40 ab	100 a	100 a	90 a	100 a	86 ab
M.26	90 a	70 a	90 a	90 a	70 ab	82 ab
M.9Pajam2	20 b	90 a	100 a	60 ab	80 ab	70 abc
G.814	80 ab	90 a	90 a	90 a	90 ab	88 a
Mean	71 c	74 bc	90 a	79 abc	85 ab	

z Cultivars and rootstocks listed in order of increasing TCSA. Letters between cultivar and rootstock means are shown following Tukey’s HSD (*P* = 0.05). Letters between rootstocks within each cultivar are shown using t-tests after a Bonferroni adjustment (*P* = 0.001).

**Figure 1:**
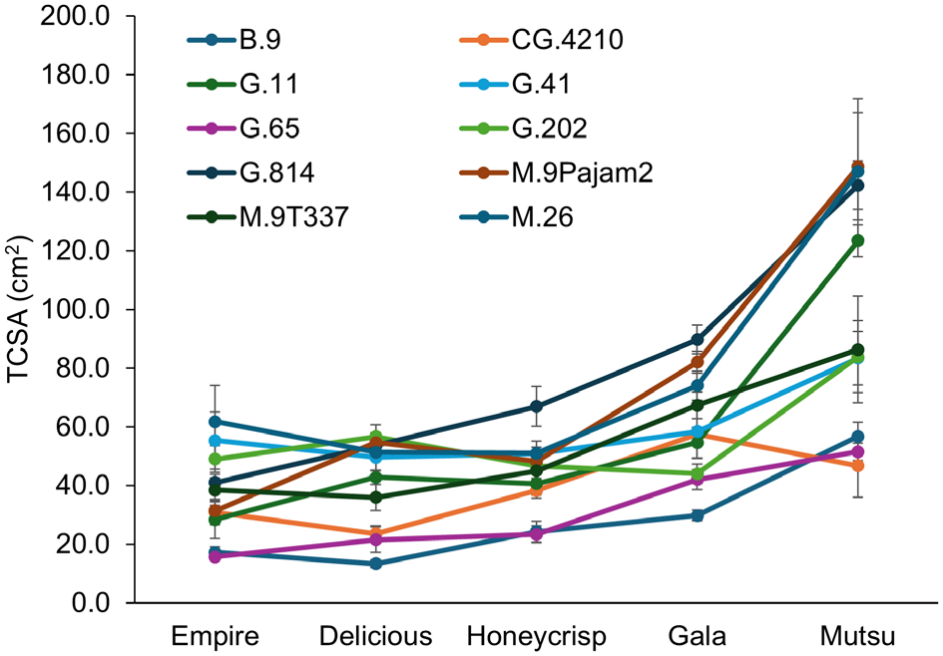
Least square mean trunk cross sectional area values (TCSA, cm^2^) by cultivar and rootstock after 17 years (2007-2023) of the dwarfing rootstocks in Geneva, NY. Error bars show standard error (n= 2-10).

There was a significant interaction (*F* = 4.1, *P* ≤ 0.001) between cultivar and rootstock for final TCSA in the dwarfing rootstock experiment (**Figure 1**; **Supplementary Table S1**). The ‘Empire’ trees were largest on G.41 and M.26 while the smallest were on B.9 and G.65. The ‘Delicious’ trees were largest on G.202 and M.9Pajam2 but smallest on B.9, G.65, and CG.4210. The ‘Honeycrisp’ trees were largest on G.814 and smallest on B.9 and G.65. The ‘Gala’ trees were largest on G.814 and smallest on B.9. The ‘Mustu’ trees on G.11, G.814, M.26, and M.9Pajam2 all had trees >100 cm^2^. The smallest ‘Mustu’ trees were B.9, CG.4210, and G.65. Across all stocks, ‘Empire’ and ‘Delicious’ trees were the smallest, followed by ‘Honeycrisp’, ‘Gala’, and finally ‘Mutsu’. Examining rootstocks across cultivars, B.9 and G.65 were consistently small, while M.26 and G.814 had the least dwarfing effect across the cultivars.

**Figure 2:**
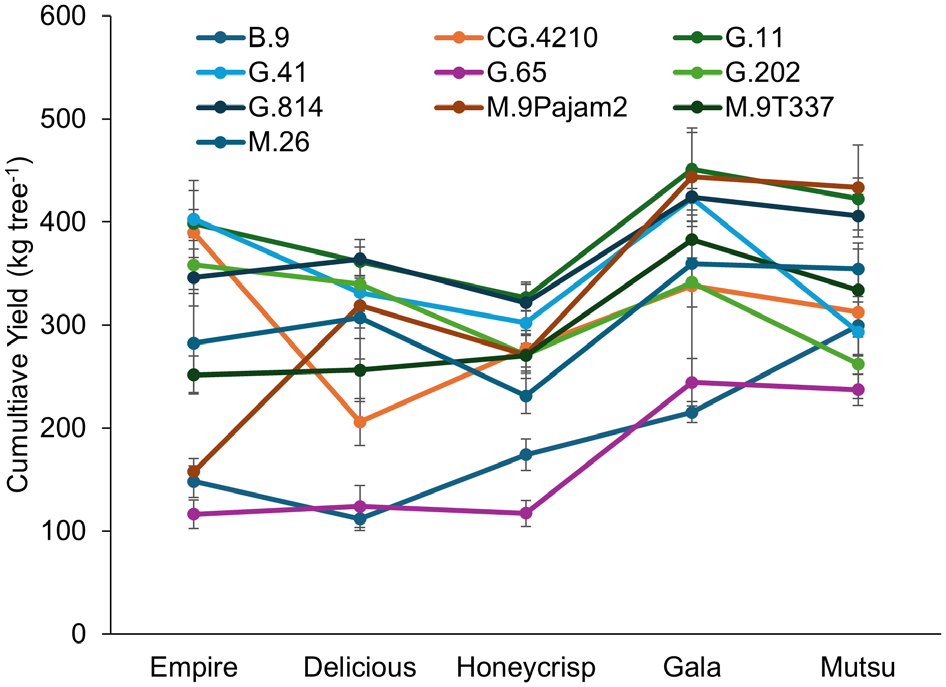
Least square mean cumulative yield values (kg tree^-1^) by cultivar and rootstock after 17 years (2007-2023) of the surviving dwarfing rootstocks in Geneva, NY. Error bars show standard error (n = 2-10).

There was a significant interaction (*F* = 3.1, *P* ≤ 0.0001) between cultivar and rootstock regarding cumulative yield in the dwarfing rootstock experiment (**Figure 2**; **Supplementary Table S2**). The ‘Empire’ cumulative yields over 350 kg tree^-1^ included G.41, CG.4210, G.11, and G.202 with the lowest yields on B.9, G.65, and M.9Pajam2. The ‘Delicious’ trees had the highest yields on G.11 and G.814 with lowest yields on B.9 and G.65. The ‘Honeycrisp’ trees on G.11 and G.814 had higher yields than B.9, G.65, and M.26. The ‘Gala’ trees had highest yields on G.11, G.41, G.814, M.9Pajam2, and M.9T337, with lowest yields on B.9 and G.65. The ‘Mutsu’ trees had highest yields on M.9Pajam2, G.11 and G.814 with the lowest yields on G.202 and G.65. While CG.4210 performed well with ‘Empire’, with all other cultivars it showed only average cumulative yields. G.41 had higher yields on all of the cultivars except for ‘Mutsu’, where it was the third lowest yielding stock. Among cultivars, the larger TCSA cultivars ‘Mustu’ and ‘Gala’ had greater cumulative yields compared to the other cultivars. Among rootstocks, G.11 had the highest cumulative yield across cultivars.

**Figure 3:**
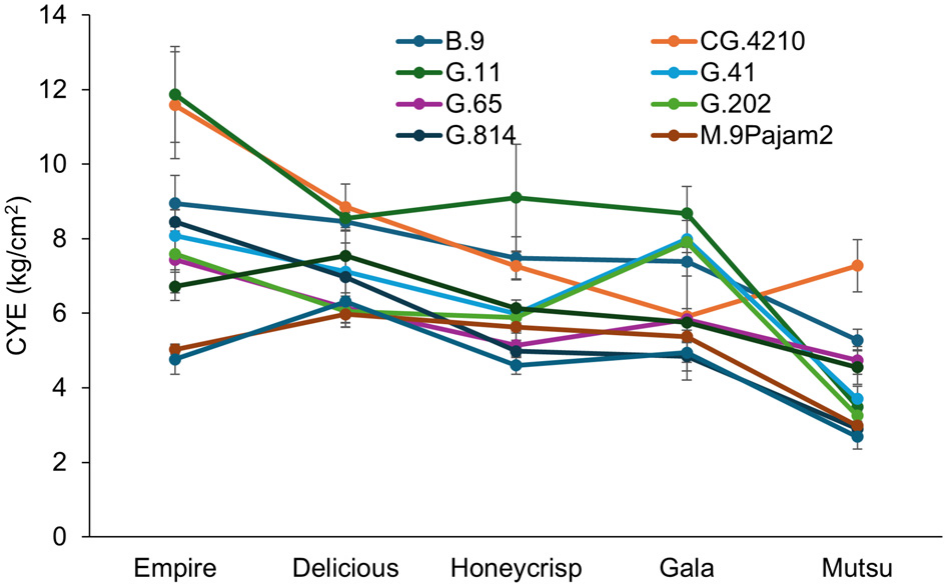
Least square mean cumulative yield efficiency values (CYE, [total kg tree^-1^]/cm^2^ final TCSA) by cultivar and rootstock after 17 years (2007-2023) of the dwarfing rootstocks in Geneva, NY (n = 2-10).

CYE also had a significant interaction (*F* = 7.5, *P* ≤ 0.001) between cultivar and rootstock in the dwarfing rootstock experiment (**Figure 3**; **Supplementary Table S3**). The ‘Empire’ trees on CG.4210 and G.11 had the highest CYE while M.26 and M.9Pajam2 had the lowest. The ‘Delicious’ trees had the highest CYE with B.9, CG.4210 and G.11 with lower CYE on G.202, G.65, M.26 and M.9Pajam2. The ‘Honeycrisp’ trees on G.11 had the highest CYE and were similar to B.9 and CG.4210 with all other stocks being statistically lower. The ‘Gala’ trees had highest CYE with G.11, G.202, and G.41 with the lowest CYE on G.814 and M.26. The ‘Mutsu’ trees on CG.4210 had the highest CYE, while G.814, M.26, and M.9Pajam2 had the lowest. Among cultivars, CYE was negatively related to increasing TCSA, and ‘Empire’ had the highest on average while ‘Mustu’ had the lowest CYE. Among rootstocks, G.11 and CG.4210 performed well, while M.26 and M.9Pajam2 had the lowest CYE.

The G.16 stock had no surviving ‘Delicious’ trees by the end of the study, and this prevented comparisons to other stocks for TCSA, cumulative yield, and CYE. There were differences by cultivar for G.16, but it followed a similar pattern as other stocks, with trees on larger rootstocks having more cumulative yield but less CYE (**Table 2**).

**Table 2:** Average trunk cross sectional area (TCSA, cm^2^), cumulative yield (kg tree^-1^), and cumulative yield efficiency (CYE, [kg tree^-1^]/cm^2^ TCSA) of the G.16 stock in the dwarfing rootstock in Geneva, NY^z^.

Interaction	TCSA	Cumulative Yield	CYE
Empire x G.16	25.0 c	202.2 c	8.3 a
Delicious x G.16	–	–	–
Honeycrisp x G.16	53.3 b	291.7 b	5.4 b
Gala x G.16	66.2 b	381.0 a	5.8 b
Mutsu x G.16	122.9 a	403.2 a	3.3 c

z Cultivars are listed in order of increasing TCSA. Different letters show mean separation test results of Tukey’s HSD (*P* ≤ 0.05).

### 3.2 Dwarfing rootstock fruit quality

**Figure 4:**
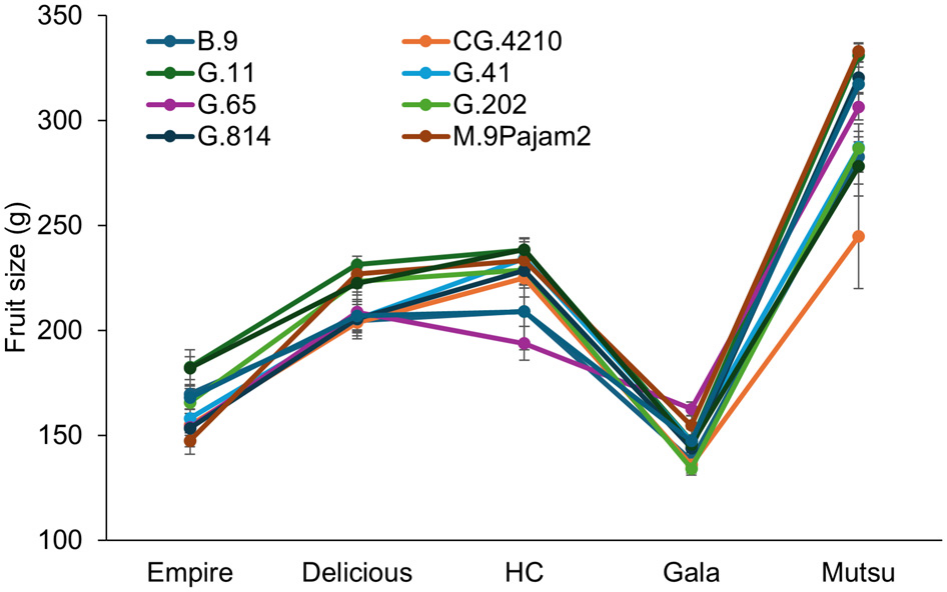
Least square mean crop load adjusted fruit size values (g) by cultivar and rootstock after 17 years (2007-2023) of the surviving dwarfing rootstocks in Geneva, NY. Error bars show standard error (n = 2-10).

The crop load adjusted fruit size also had a significant interaction (*F* = 4.1, *P* ≤ 0.0001) between cultivar and rootstock (**Figure 4**; **Supplementary Table S4**). Only ‘Empire’ and ‘Gala’ trees had significant differences between rootstocks. The ‘Empire’ trees had larger fruit size on G.11, which was approximately 40g larger than G.65, G.814, and M.9Pajam2. The ‘Gala’ trees had larger fruit on G.65 and smaller sizes were found on B.9 and G.202. Among cultivars, the largest fruit size was with ‘Mutsu’, while the smallest fruit was with ‘Gala’. Among rootstocks, G.11 had the highest fruit size on average, while CG.4210 had the smallest fruit size.

There were no significant interactions between cultivar and rootstock for L/D ratio, fruit firmness, or soluble solids in the dwarfing rootstock experiment (**Table 3**). The cultivar effect was significant (*F* = 33.3, *P* ≤ 0.0001) for L/D ratio, as ‘Honeycrisp’ has a lower ratio in comparison to the other cultivars. Both ‘Empire’, ‘Delicious’, and ‘Gala’ had lower firmness (*F* = 27.7, *P* ≤ 0.0001) than ‘Honeycrisp’ and ‘Mustu’. There was a significant effect of cultivar on soluble solids (*F* = 12.3, *P* ≤ 0.0001) as ‘Mutsu’ had higher °brix than the other cultivars. There was also a significant effect of rootstock on soluble solids across cultivars (*F* = 3.2, *P* ≤ 0.001) as B.9, G.41, and M.26 had higher °brix than G.11.

**Table 3:** Least square mean length diameter ratio (L/D), firmness (lbs), and soluble solids values (°brix) of the dwarfing rootstocks between the five cultivars (n = 6-8)^z^.

Rootstock	Empire	Delicious	Honeycrisp	Gala	Mutsu	Mean
L/D						
B.9	0.93	0.95	0.85	0.96	0.94	0.93
G.65	0.95	0.94	0.83	0.96	0.94	0.92
CG.4210	0.95	0.96	0.84	0.97	0.94	0.93
M.9T337	0.95	0.96	0.84	0.98	0.95	0.94
G.202	0.94	0.95	0.86	0.97	0.95	0.93
G.41	0.95	0.94	0.85	0.94	0.96	0.93
G.11	0.94	0.95	0.86	0.97	0.94	0.93
M.26	0.93	0.98	0.85	0.99	0.97	0.94
M.9Pajam2	0.96	0.96	0.85	0.96	0.95	0.94
G.814	0.94	0.99	0.86	0.97	0.96	0.94
Mean	0.94 a	0.96 a	0.85 b	0.97 a	0.95 a	
Firmness (lbs)						
B.9	12.50	12.58	14.46	14.08	14.76	13.68
G.65	12.34	11.72	15.2	13.48	14.73	13.49
CG.4210	12.55	12.47	15.33	13.07	14.73	13.63
M.9T337	12.51	13.06	14.18	14.57	14.78	13.82
G.202	12.57	12.79	14.86	14.07	15.48	13.95
G.41	12.19	13.28	14.82	12.97	14.99	13.65
G.11	12.57	12.85	14.09	13.18	14.59	13.46
M.26	12.11	12.86	14.44	14.29	14.21	13.59
M.9Pajam2	11.97	12.59	14.66	13.55	15.08	13.57
G.814	12.88	13.34	14.44	13.57	14.56	13.76
Mean	12.41 c	12.75 c	14.65 a	13.68 b	14.79 a	
Soluble Solids (°brix)						
B.9	13.35	13.41	13.5	13.6	14.45	13.66 a
G.65	13.48	13.75	13.29	12.58	13.96	13.41 ab
CG.4210	12.46	13.23	13.06	12.35	13.59	12.94 ab
M.9T337	13.01	13.05	13.37	13.78	14.18	13.48 a
G.202	13.03	13.66	13.67	13.55	14.35	13.65 a
G.41	12.82	13.01	13.64	12.53	14.21	13.24 ab
G.11	11.43	13.08	12.84	12.37	13.63	12.67 b
M.26	13.01	12.98	13.08	13.25	14.03	13.27 ab
M.9Pajam2	13.00	13.00	13.30	13.18	14.15	13.32 ab
G.814	13.05	13.72	12.86	12.78	13.45	13.17 ab
Mean	12.86	13.29	13.26	13.00	14.00	

z Cultivars and rootstocks listed in order of increasing TCSA. Different letters show differences of the main effects by Tukey’s HSD test (*P* = 0.05).

The three bicolor cultivar color analysis also showed significant interactions between cultivar and rootstock (**Table 4**). Green color was higher on ‘Honeycrisp’ (*F* = 64.0, *P* ≤ 0.0001) compared to ‘Empire’ and ‘Gala’ but no differences were found between rootstocks across cultivars and no interaction occurred. There was more yellow color on ‘Honeycrisp’ compared to the other two bi-color cultivars (*F* = 95.8, *P* ≤ 0.0001). Among rootstocks, CG.4210 had the highest yellow color, while the lowest numerical value was found on M.26 (*F* = 4.9, *P* ≤ 0.0001). There was also a significant interaction of yellow color (*F* = 2.1, *P* ≤ 0.01). The ‘Empire’ trees had high yellow color with CG.4210 and G.11 while G.65 had less yellow color, no differences were found within ‘Honeycrisp’ between rootstocks, and the ‘Gala’ trees had more yellow color on CG.4210, which was higher than all other stocks except for G.41. The significant interaction (*F* = 2.6, *P* ≤ 0.01) was also present for red color between cultivar and rootstock. Within individual cultivars, average red color of ‘Empire’ was highest on G.65 and lowest on CG.4210. The ‘Honeycrisp’ trees had the highest red color on G.202 and similar low red color on G.65 and M.9Pajam2. The ‘Gala’ trees had consistent red color across all stocks except for CG.4210. Among cultivars, the red color was highest on ‘Empire’ and ‘Gala’ compared to ‘Honeycrisp’ (*F* = 160.0, *P* ≤ 0.0001). Among rootstocks, M.26 had the highest red color with CG.4210, G.41, and G.11 had the least (*F* = 5.6, *P* ≤ 0.0001).

**Table 4:** Least square mean values (%) of green, yellow, and red color between the dwarfing rootstocks and the three bi-color apples in the study (n = 6-8)^z^.

Rootstock	Empire	Honeycrisp	Gala	Mean
Green (%)				
B.9	0.6	4.9	0.9	2.3
G.65	0.5	9.7	0.8	3.7
CG.4210	1.5	6.9	1.2	3.0
M.9T337	1.0	4.8	1.1	2.5
G.202	1.0	2.8	0.9	1.7
G.41	1.2	4.7	0.9	2.4
G.16	0.8	4.0	1.3	2.0
G.11	1.4	12.1	0.9	5.1
M.26	0.6	5.0	0.8	2.3
M.9Pajam2	1.0	10.8	1.1	4.5
G.814	0.8	5.7	0.8	2.6
Mean	0.9 b	7.1 a	1.0 b	
Yellow (%)				
B.9	31.2 de	53.1 a	38.9 b	41.3 bc
G.65	26.6 e	59.9 a	39.7 b	42.1 bc
CG.4210	52.4 a	59.4 a	57.6 a	55.8 a
M.9T337	40.0 abcde	55.3 a	34.4 b	43.5 bc
G.202	32.8 cde	50.6 a	39.6 b	41.2 bc
G.41	45.8 abc	57.2 a	44.7 ab	49.4 ab
G.16	34.0 cde	54.7 a	35.8 b	41.5 bc
G.11	48.1 ab	57.3 a	38.3 b	48.4 abc
M.26	30.6 de	53.7 a	36.6 b	40.5 c
M.9Pajam2	34.6 bcde	58.8 a	36.8 b	43.6 bc
G.814	42.7 abcd	51.9 a	38.0 b	44.4 bc
Mean	38.1 b	55.8 a	39.8 b	
Red (%)				
B.9	68.2 ab	42.1 ab	60.1 a	56.4 ab
G.65	72.9 a	30.4 b	59.5 a	54.2 abc
CG.4210	46.1 e	33.6 ab	41.3 b	41.2 d
M.9T337	59.0 abcde	39.9 ab	64.4 a	54.1 abc
G.202	66.2 abc	46.7 a	59.5 a	57.1 ab
G.41	53.0 cde	38.1 ab	54.5 ab	48.2 bcd
G.16	65.3 abc	41.3 ab	62.9 a	56.5 ab
G.11	50.5 de	30.6 ab	60.8 a	46.5 cd
M.26	68.9 ab	41.3 ab	62.6 a	57.2 a
M.9Pajam2	64.4 abcd	30.4 b	62.1 a	52.0 abcd
G.814	56.5 bcde	42.4 ab	61.2 a	53.0 abc
Mean	61.0 a	37.1 b	59.2 a	

z Cultivars and rootstocks listed in order of increasing TCSA. Letters between cultivar and rootstock means are shown following Tukey’s HSD (*P* = 0.05). Letters between rootstocks within each cultivar are shown using t-tests after a Bonferroni adjustment (*P* = 0.001).

### 3.3 Semi-dwarfing rootstock horticultural performance

The wider orchard spacing semi-dwarfing rootstock experiment also had multiple horticultural parameters which showed significant interactions between scion and rootstock, but smaller *F* values in comparison to the first experiment which suggests less differences between treatment comparisons. Nonetheless, the average survival showed a significant interaction (*F* = 2.7, *P* ≤ 0.0001) of cultivar and rootstock (**Table 5**). Stocks with ≥ 90% survival included G.222, G.30, and M.7 with ‘Delicious’; all except M.7 with ‘Honeycrisp’; B.118, G.222, G.30, and M.26 with ‘Empire’; B.118, G.214, M26, and M.7 with ‘Gala’; and B.118, G.214, G.222, M.26, and M.7 with ‘Mutsu’. Among cultivars the highest rate of survival was ‘Honeycrisp’ and the lowest were ‘Delicious’ and ‘Gala’. Among rootstocks, B.118, G.222, and M.26 all had ≥ 90% survival by the conclusion of the study. All cultivars showed the worst survival with G.210.

**Table 5:** Average survival (%) by cultivar and rootstock after 17 years (2007-2023) of the semi-dwarfing rootstocks in Geneva, NY (n = 1-10)^z^.

Rootstock	Empire	Delicious	Honeycrisp	Gala	Mutsu	Mean
G.935	80 ab	90 a	80 a	70 ab	80 ab	80 b
G.214	70 ab	100 a	80 a	100 a	90 ab	88 ab
M.26	80 ab	90 a	100 a	90 a	90 ab	90 ab
G.30	100 a	100 a	100 a	60 ab	80 ab	88 ab
G.222	100 a	100 a	100 a	60 ab	100 a	92 ab
G.210	40 b	100 a	10 b	30 b	50 b	46 c
M.7	100 a	80 a	60 a	100 a	90 ab	86 ab
B.118	80 ab	100 a	100 a	100 a	100 a	96 a
Mean	81 ab	95 a	79 b	76 b	85 ab	

z Cultivars and rootstocks listed in order of increasing TCSA. Letters between cultivar and rootstock means are shown following Tukey’s HSD (*P* = 0.05). Letters between rootstocks within each cultivar are shown using t-tests after a Bonferroni adjustment (*P* = 0.002).

**Figure 5:**
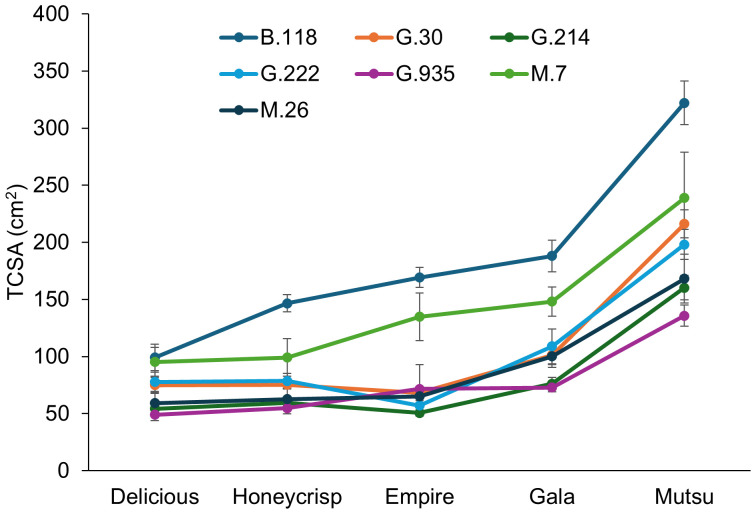
Least square mean trunk cross sectional area (TCSA) values (cm^2^) by cultivar and rootstock after 17 years (2007-2023) of the semi-dwarfing rootstocks in Geneva, NY. Error bars show standard error (n = 6-10).

The TCSA of the semi-dwarfing experiment also had a significant interaction (*F* = 2.6, *P* ≤ 0.001) between cultivar and rootstock (**Figure 5**; **Supplementary Table S5**). The largest stocks (> 90 cm^2^) were B.118 and M.7 for ‘Delicious’ trees, while G.935 had less than 50 cm^2^. ‘Honeycrisp’ trees on B.118 were the largest with over 140 cm^2^, while G.214 and G.935 had the smallest size under 60 cm^2^. The ‘Empire’ trees were largest on B.118 and M.7 (>130 cm^2^), with the smallest trees (< 50 cm^2^) on G.214. Unlike other cultivars which had G.935 as one of the smallest stocks, it was numerically the third largest with ‘Empire’. The ‘Gala’ trees on B.118 were largest (>175 cm^2^), while G.214 and G.935 had the smallest size (< 80 cm^2^). The ‘Mutsu’ trees were largest on B.118 at over 300 cm^2^ on average, with the smallest trees on G.214 and G.935. At the larger orchard spacing of the semi-dwarf experiment, the order of average tree size by cultivar was different in comparison to the dwarfing rootstock experiment. In the semi-dwarfing experiment, the ‘Delicious’ and ‘Honeycrisp’ trees were the smallest across rootstocks, with increasing size for ‘Empire’, ‘Gala’, and finally largest with ‘Mutsu’. The largest rootstocks across cultivars were B.118 and M.7.

**Figure 6:**
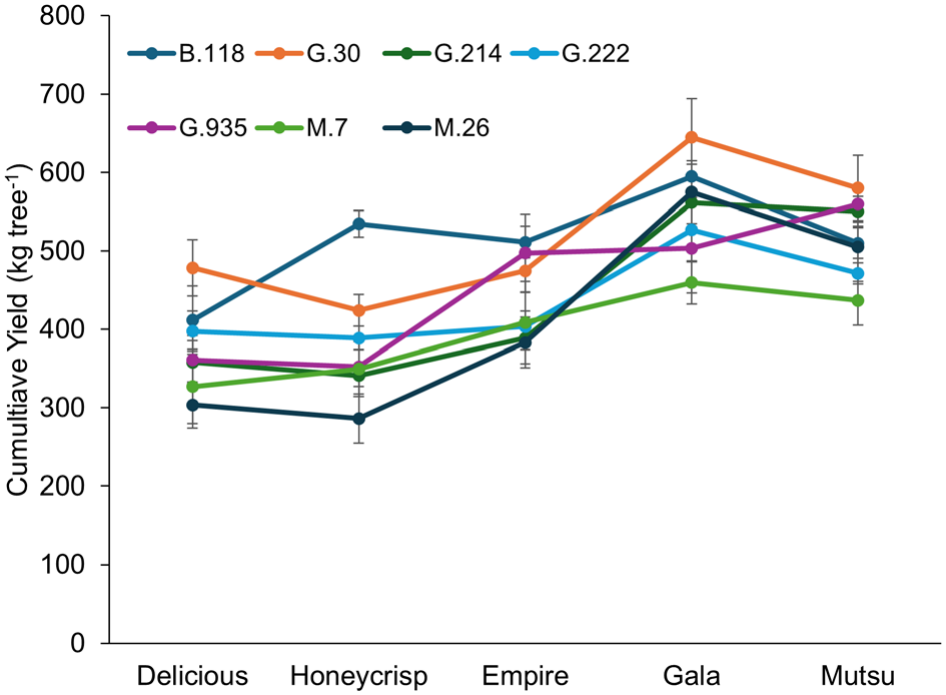
Least square mean cumulative yield (kg tree^-1^) by cultivar and rootstock after 17 years (2007-2023) of the dwarfing rootstocks in Geneva, NY. Error bars show standard error (n = 6-10).

Cumulative yield from the semi-dwarfing experiment also had a significant interaction (*F* = 2.1, *P* ≤ 0.01) between cultivar and rootstock (**Figure 6**; **Supplementary Table S6**). The interaction examined with the mean comparison within each cultivar only suggested differences for ‘Delicious’ and ‘Honeycrisp’ trees. The G.30 stock had the highest cumulative yield with ‘Delicious’, while M.26 and M.7 had lower yields. The ‘Honeycrisp’ trees on B.118 had the highest yields, with lowest yields on M.26. The largest cumulative yield was observed with ‘Gala’ trees, while ‘Delicious’ had the smallest cumulative yield across stocks. The highest cumulative yield from any stock across cultivar was G.30, while M.26 and M.7 had the lowest.

**Figure 7:**
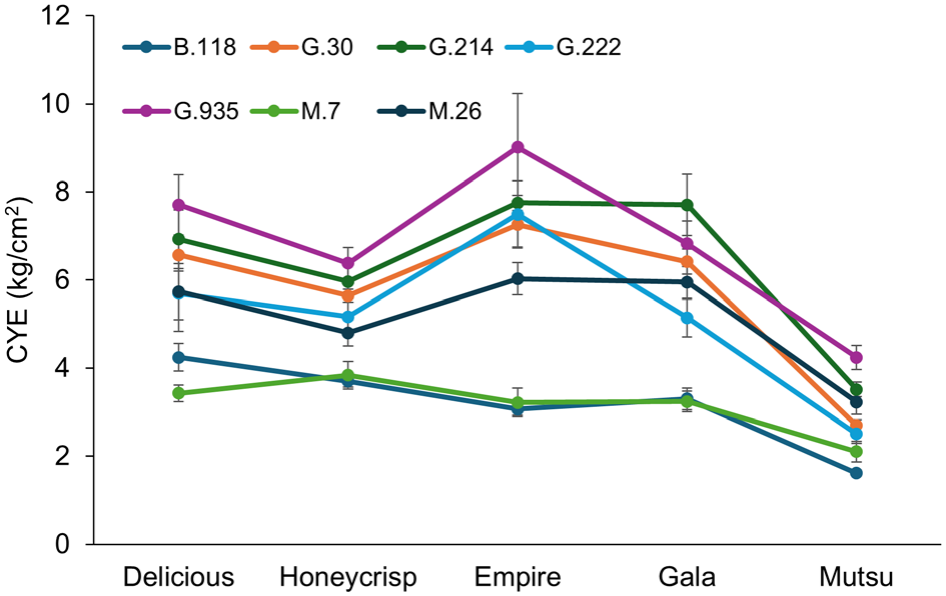
Least squared mean cumulative yield efficiency values ([total kg tree^-1^]/cm^2^ final TCSA) by cultivar and rootstock after 17 years (2007-2023) of the semi-dwarfing rootstocks in Geneva, NY (n = 6-10).

There was a significant interaction (*F* = 2.1, *P* ≤ 0.001) between cultivar and rootstock in the semi-dwarfing experiment regarding CYE (**Figure 7**; **Supplementary Table S7**). The highest CYE within cultivars was usually associated with the smallest average TCSA, G.935, with the lowest CYE on stocks with the highest TCSA, M.7 and B.118. However, the ‘Gala’ trees had the highest CYE on G.214 and G.222 had similar CYE to G.935 for all the cultivars except ‘Mutsu’. Among cultivars, ‘Empire’ had the highest CYE while ‘Mustu’ had the lowest. Among rootstocks, G.935 had the highest CYE in the semi-dwarfing rootstocks, while B.118 and M.7 had the lowest CYE values.

The G.210 stock had only one surviving ‘Empire’ tree by the end of the study and this prevented comparisons to other stocks for TCSA, cumulative yield, and CYE. The significant cultivar differences for G.210 had a similar pattern according to TCSA, with the cultivar with the largest trees (‘Mutsu’) having more cumulative yield, and less CYE (**Table 6**).

**Table 6:** Average trunk cross sectional area (TCSA, cm^2^), cumulative yield (kg/tree^-1^), and cumulative yield efficiency (CYE, [kg tree^-1^]/cm^2^ TCSA) of G.210 in the semi-dwarfing rootstock experiment in Geneva, NY^z^.

Interaction	TCSA	Cumulative yield	CYE
Delicious x G.210	75.7 c	411.2 c	5.7 a
Honeycrisp x G.210	95.7 bc	480.2 bc	5.1 a
Empire x G.210	–	–	–
Gala x G.210	133.0 b	703.3 a	5.5 a
Mutsu x G.210	234.1 a	606.4 ab	2.7 b

z Cultivars are listed in order of increasing TCSA. Different letters show mean separation test results of Tukey’s HSD (*P* ≤ 0.05).

### 3.4 Semi-dwarfing rootstock fruit quality

**Figure 8:**
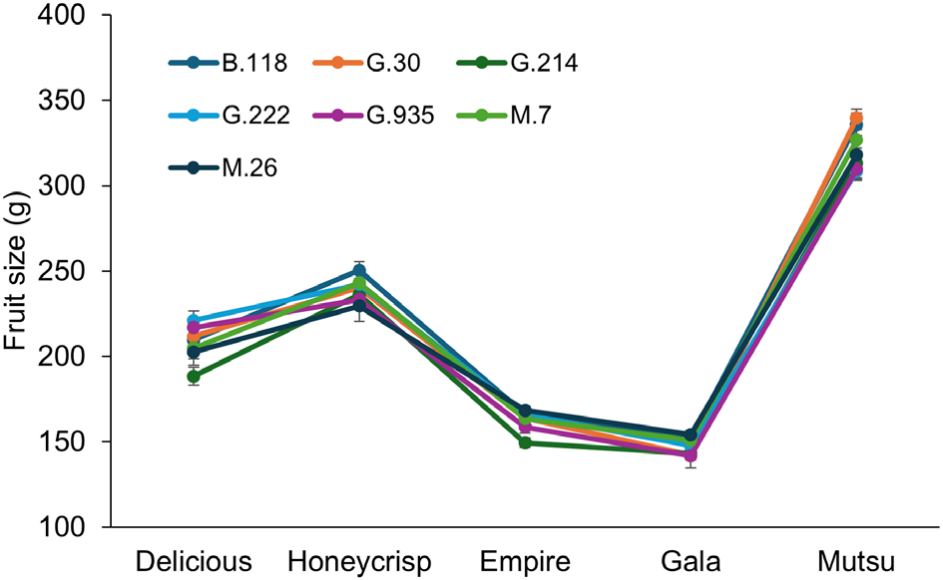
Least squared mean crop load adjusted fruit size values (g) by cultivar and rootstock after 17 years (2007-2023) of the semi-dwarfing rootstocks in Geneva, NY (n = 6-10).

There was no significant interaction between cultivar and rootstock regarding crop load adjusted fruit size in the semi-dwarfing experiment (**Figure 8**; **Supplementary Table S8**). There were differences in crop load adjusted fruit size between the cultivars (*F* = 80.0, *P* ≤ 0.0001), with ‘Gala’ having the smallest size fruit followed by ‘Empire’, ‘Delicious’, ‘Honeycrisp’, and finally ‘Mutsu’. There were no differences between rootstocks across cultivars.

There were no significant interactions between cultivar and rootstock regarding fruit L/D ratio, firmness, or soluble solids in the semi-dwarfing experiment (**Table 7**). There were significant differences between cultivars for L/D ratio (*F* =25.9, *P* ≤ 0.0001), as ‘Honeycrisp’ had a lower ratio than the other cultivars. Fruit firmness also had differences between cultivars as ‘Honeycrisp’ and ‘Mutsu’ were higher than ‘Gala’; while all three were higher than ‘Delicious’ and ‘Empire’ (*F* =16.8, *P* ≤ 0.0001). The ‘Delicious’, ‘Honeycrisp’ and ‘Mutsu’ trees had higher soluble solids than ‘Gala’ across rootstocks (*F* =15.9, *P* ≤ 0.0001). There were no differences between stocks across cultivars for L/D ratio, firmness, or soluble solids.

**Table 7:** Least square means of length diameter ratio (L/D), firmness (lbs), and soluble solids (°brix) values of the semi-dwarfing rootstocks between the five cultivars (n = 6-8)^z^.

Rootstock	Empire	Delicious	Honeycrisp	Gala	Mutsu	Mean
L/D						
G.935	0.95	0.84	0.92	0.95	0.94	0.92
G.214	0.95	0.85	0.96	0.95	0.95	0.93
M.26	0.95	0.84	0.93	0.96	0.97	0.93
G.30	0.96	0.85	0.95	0.97	0.96	0.94
G.222	0.96	0.85	0.95	0.96	0.96	0.94
G.210	0.96	0.85	0.93	0.98	0.95	0.93
M.7	0.96	0.85	0.94	0.96	0.95	0.93
B.118	0.97	0.86	0.95	0.95	0.96	0.94
Mean	0.96 a	0.85 b	0.94 a	0.96 a	0.95 a	
Firmness (lbs)						
G.935	12.56	14.80	12.86	13.06	14.27	13.51
G.214	12.65	14.88	12.55	13.99	14.52	13.72
M.26	12.66	14.67	12.25	13.04	14.30	13.38
G.30	12.51	14.80	12.67	13.52	14.60	13.62
G.222	12.83	15.52	12.33	12.95	14.37	13.60
G.210	12.05	14.25	12.36	13.15	14.72	13.31
M.7	12.32	13.50	12.17	13.39	14.40	13.16
B.118	12.47	14.10	12.34	13.72	15.18	13.56
Mean	12.51 c	14.56 a	12.44 c	13.35 b	14.54 a	
Soluble Solids (°brix)						
G.935	13.74	13.4	12.93	12.97	13.52	13.31
G.214	13.26	13.65	12.83	13.11	13.89	13.35
M.26	13.46	13.20	13.06	12.21	13.72	13.13
G.30	13.43	13.64	12.60	12.51	13.65	13.17
G.222	13.39	13.73	12.78	12.30	13.73	13.18
G.210	13.68	13.34	13.34	12.05	13.60	13.20
M.7	13.63	13.14	12.74	12.48	13.62	13.12
B.118	13.38	12.88	12.95	12.47	13.40	13.02
Mean	13.50 a	13.37 ab	12.90 bc	12.51 c	13.64 a	

z Cultivars and rootstocks listed in order of increasing TCSA. Different letters show differences of the main effects by Tukey’s HSD test (*P* = 0.05).

The three bi-color cultivars in the semi-dwarf rootstock experiment also showed significant interactions for color (**Table 8**). There was a significant interaction between the two main effects for green color (*F* = 2.2, *P* ≤ 0.01). Within cultivars, the ‘Honeycrisp’ trees had highest green color on G.210, which was higher than G.30, G.222, M.7, and B.118. No differences of green color were found between the stocks for ‘Empire’. For ‘Gala’, G.210 had the highest green color while G.935, M.26, and B.118 had less. Among cultivars, the green color was higher on ‘Honeycrisp’ than ‘Empire’ and ‘Gala’ (*F* = 29.1, *P* ≤ 0.0001). Among rootstocks, green color was highest on G.210 and lower on G.222 and B.118 (*F* = 2.0, *P* ≤ 0.05). Yellow color also had a significant interaction between cultivar and rootstock (*F* = 2.0, *P* ≤ 0.05). While there were no rootstock differences within ‘Honeycrisp’ or ‘Empire’, the ‘Gala’ trees had higher yellow color on G.210 compared to G.935, M.26, M.7 and B.118. Between cultivars, yellow color was highest for ‘Honeycrisp’ followed by ‘Gala’ and then ‘Empire’ (*F* = 88.6, *P* ≤ 0.0001). No rootstock differences were observed among cultivars for yellow color. The model also suggested a significant interaction between cultivar and rootstock for red color (*F* = 1.9, *P* ≤ 0.05). Like the yellow color, both ‘Honeycrisp’ and ‘Empire’ had no differences of color between stocks, but ‘Gala’ red color was highest on G.935 and M.7 with less red color found on G.222 and G.210. Cultivars were significantly different (*F* = 104.5, *P* ≤ 0.0001) with ‘Empire’ having more red color than ‘Gala’, and both were higher than ‘Honeycrisp’. The significant effect of rootstock (*F* = 2.4, *P* ≤ 0.05) across the cultivars showed M.26 and M.7 with higher red color than G.210.

**Table 8:** Least square mean values (%) of green, yellow, and red color in the semi-dwarf rootstock experiment between the three bi-color apple cultivars (n = 6-8)^z^.

Rootstock	Honeycrisp	Empire	Gala	Mean
Green (%)				
G.935	2.7 ab	0.9 a	0.6 b	1.4 ab
G.214	2.9 ab	0.7 a	0.8 ab	1.5 ab
M.26	3.2 ab	0.5 a	0.6 b	1.4 ab
G.30	2.1 b	0.9 a	0.9 ab	1.3 ab
G.222	2.0 b	0.3 a	0.8 ab	1.1 b
G.210	7.7 a	0.6 a	1.1 a	3.1 a
M.7	2.3 b	1.3 a	0.8 ab	1.4 ab
B.118	1.9 b	0.8 a	0.7 b	1.1 b
Mean	3.1 a	0.7 b	0.8 b	
Yellow (%)				
G.935	56.2 a	39.2 a	32.6 c	43.2 a
G.214	58.8 a	37.5 a	43.5 abc	46.6 a
M.26	55.8 a	32.5 a	33.8 bc	41.2 a
G.30	58.0 a	35.5 a	41.2 abc	44.9 a
G.222	58.5 a	24.1 a	47.4 ab	43.3 a
G.210	62.1 a	38.6 a	51.7 a	50.8 a
M.7	57.1 a	34.5 a	32.8 c	41.5 a
B.118	62.0 a	35.8 a	37.8 bc	44.7 a
Mean	58.2 a	34.3 c	39.6 b	
Red (%)				
G.935	41.1 a	59.7 a	66.7 a	55.4 ab
G.214	38.3 a	61.8 a	55.7 abc	52 ab
M.26	41.1 a	66.9 a	65.6 ab	57.5 a
G.30	39.8 a	63.4 a	57.9 abc	53.8 ab
G.222	39.5 a	75.6 a	51.8 bc	55.6 ab
G.210	30.1 a	60.8 a	47.2 c	46.1 b
M.7	40.6 a	64.2 a	66.4 a	57.1 a
B.118	36.2 a	63.4 a	61.6 ab	54.2 ab
Mean	38.7 c	64.9 a	59.6 b	

z Cultivars and rootstocks listed in order of increasing TCSA. Letters between cultivar and rootstock means are shown following Tukey’s HSD (*P* = 0.05). Letters between rootstocks within each cultivar are shown using t-tests after a Bonferroni adjustment (*P* = 0.002).

## 4 Discussion

Following 17-years of growth, each of the horticultural variables measured showed a significant interaction between cultivar and rootstock in both the dwarfing and the semi-dwarfing rootstock orchards. Identification of cultivar and rootstock interactions have been identified previously by Autio et al. (2001) and Reig et al. (2019a). Autio et al. (2001) reported that the interactions become less important as trees age; however, the current work which identified interactions after a longer period of time suggests that the interactive effects would result in potential benefits or opportunity costs for growers if they were to pair multiple cultivars on less performing rootstock or visa-versa. Reig et al. (2019a) reported after 11 years a significant interaction caused by B.9 and G.41 which had high yield performance with ‘Gala’ and ‘Honeycrisp’ cultivars but the lowest performance with ‘Fuji’.

Unlike Autio et al. (2001), which did not report a significant interaction between cultivar and rootstock for tree survival, both experiments in the current study had an interaction between cultivar and rootstock. Apart from land cost, a large expense for growers during establishment are trees themselves, both at time of planting and as replants (Robinson et al., 2007). Several cultivar and rootstock pairings in our study showed very high mortality with over 50% tree death after 17 years. Two stocks showed very high mortality generally across cultivars, such as CG.4210 in the dwarfing experiment and G.210 for the semi-dwarf experiment, however ‘Honeycrisp’ had 100% survival on both stocks after 17 years. Such inconsistencies in mortality were apparent in other cultivar and rootstock pairings. Using this study as a guide, growers in a similar growing region probably should avoid using G.16 paired with ‘Delicious’, CG.4210 with ‘Gala’, M.9Pajam2 with ‘Empire’ but not necessarily avoid the same stocks for other cultivars. In previous studies, G.16 stocks have been known to be sensitive to viruses transferred from the scion (Robinson et al., 2003) and this may account for why the ‘Delicious’ trees 100% tree death. The unreleased CG.4210 stock and the M.9Pajam2 trees had very high mortality following the first several years of establishment, possibly due to fire blight and replant issues. The 2010 NC-140 ‘Honeycrisp’ studies spans including a 5-year (Autio et al., 2017) and 8-year (Autio et al., 2020) report show different results in comparison. For example, ‘Honeycrisp’ after 8 years in New York conditions had 100% survival. In the current study, only 60% survival occurred on B.9 after 17 years. While a small sample size of ten trees in our study could be seen as a possible limitation, other similar pairings with ‘Honeycrisp’ after 8-years, G.11 (100%), G.202 (80%), and M.9T337 (100%) in the 8-year ‘Honeycrisp’ trial (Autio et al., 2020) match the survival ratings reported in our study. A ten-year study across multiple locations did not find a significant interaction between cultivars and stocks (Autio et al., 2001) and instead was largely influenced by cultivar susceptibility to fire blight, which occurred within the present study but was managed quickly to avoid entire tree death. Unlike the dwarfing experiment, no single cultivar and rootstock combination yielded greater than 50% mortality, except for 4 out of the 5 cultivars on G.210 in the semi-dwarfing experiment. The lower survival of G.210 stock except for ‘Honeycrisp’ was unexpected, as the stock generally has survived well in multiple locations (Robinson et al., 2014). High mortality of the cultivars other than ‘Honeycrisp’ is possibly due to a combination of factors, including latent virus susceptibility of the G.210 stock, as it shares similar lineage of G.935 (Fazio et al., 2022), and fire blight, since ‘Honeycrisp’ has alleles which convey reduced susceptibility to the pathogen (Kostick et al., 2021). The Bonferroni adjustment conveys a more conservative estimate of rootstock differences within cultivars and few other cultivar and rootstock pairings had statistically lower survivability than G.210. Nonetheless, growers may consider avoiding planting several pairings as a result of this study, including ‘Gala’ on G. 222 or G.30, as well as ‘Empire’ on M.7, as they each had 40% mortality.

The TCSA comparisons after 17 years also resulted in significant interactions between cultivar and rootstock for both experiments. In the semi-dwarfing experiment, a general pattern of rootstock vigor was apparent within each cultivar although there was still a significant interaction. These results agree with a previous 10-year report which identified a similar TCSA interaction but found consistent differences between stocks within the cultivars studied (Autio et al., 2001). In contrast, the dwarfing experiment interaction for TCSA showed greater differences between cultivars, and inconsistent relative size of stocks such as G.202 for ‘Delicious’, M.9Pajam2 for ‘Honeycrisp’, and ‘CG.4210’ for ‘Mutsu’ accounted for a large proportion of the interactive effect. One possible source of variation which could explain the TCSA interaction for both orchard spacings would be increased growth of trees adjacent to gaps or missing trees which had died during the study. However, adding a covariate into the model to account for proximity to missing trees, which may provide a growth advantage with added sun interception or nutrients from less root competition, did not account for the interactions observed (data not shown). With increasing costs of production and labor, growers often are employing measures to reduce tree vigor. While scion and stock interactions are being explored at the molecular level (Tworkoski and Fazio, 2016), our study showed how these mechanisms can manifest over an orchard lifetime and should be considered when planning future orchards.

The cumulative yield interaction between rootstock and cultivar followed a similar pattern to TCSA. While the smallest TCSA stocks generally had the highest CYE while the larger TCSA stocks had lower CYE in both orchard spacings, a few exceptions occurred. Rootstocks B.9 and G.11 were most likely to have the highest CYE in the dwarfing rootstock experiment regardless of cultivar. This was due to the small TCSA size of the trees after 17 years, and productive nature of the stocks as a function of hormonal differences resulting in flatter branch angle (Lordan et al., 2017). However, trees on B.9 and G.11 often failed to grow to the top wire without filling the available space for light capture, which could be a substantial opportunity cost for growers (Fazio and Robinson, 2021). The CG.4210 stock often had very high CYE values, matching B.9 and G.11. In a 4-year study which examined tree growth with or without preplant fumigation, CG.4210 also had one of the highest CYE values in comparison to other stocks (Robinson et al., 2012). While the CG.4210 stock performed well, the high mortality in the study would make it unsuitable for growers, although further evaluations are needed for ‘Honeycrisp’ as the stock had 100% survival. The larger dwarfing stocks also showed inconsistent trends. For example, G.814 frequently had low CYE values with ‘Honeycrisp’, ‘Gala’, and ‘Mustu’, but the stock was not the lowest CYE for ‘Empire’ and ‘Delicious’. A previous study with ‘Fuji’ in the Hudson Valley region of New York, USA, reported low CYE after 11 years for G.814, but this could be a result of the vigorous ‘Fuji’ scion. Apart from tree size, crop load of the trees strongly influences not only current season yield but the subsequent return bloom (Campbell and Kalcsits, 2024). Previous work with ‘Honeycrisp’ showed that G.11 and G.814 had high yields and good return bloom (Lordan et al., 2017) which matches our dwarfing rootstock experiment, as both G.11 and G.814 had the highest cumulative yields across cultivars. Cultivars did vary in fruit size in both studies, but the conservative threshold of rootstock differences within cultivars only resulted in differences for ‘Empire’ and ‘Gala’ in the dwarfing study.

Previous cultivar and rootstock interactions have been observed influencing tree size due to hormone concentrations (Tworkoski and Fazio, 2016) and hormonal profile differences result in different tree behaviors (Lordan et al., 2017). Tree roots represent the ‘unseen’ portion of the tree and while research on the effects of different scions on root growth is lacking in the literature, there have been examples that have described significant effects of different scions on graft union formation (Adams et al., 2018), suggesting the possibility that such changes might be mirrored below ground. The interplay of the scion and rootstock along with the hormonal profile determines soil water and nutrient acquisition, leading to fruit quality differences, such as fruit size and rates of bitter pit (Lordan et al., 2017; Valverdi and Kalcsits, 2021). However, in both experiments of this study, the cultivar (scion) differences were by far the most important factors in the model, with less differences found between rootstocks across cultivars and no significant interactions occurring. Fruit yellow and red color in the dwarfing experiment, and all three colors measured in the semi-dwarfing experiment resulted in significant interactions, however these differences were minor in comparison to the scion effect. The semi-dwarfing experiment had the largest color differences for ‘Gala’, where G.210 had the least red color. Regardless, thresholds of red color which determine higher price point for growers should be considered when examining interaction studies in the future.

While long-term interactions between cultivar and rootstocks are interesting to analyze scientifically, providing economic justification for a scion-rootstock combination over another would be very helpful for growers. Our current study did not use economic tools and examining how interactions regarding tree mortality, cumulative yields, or fruit size would influence long-term profitability is needed. Different rootstocks depending on the training system can have different economic returns (Ho et al., 2024), and it is possible that horticulturally superior scion-rootstock pairings identified in this study would not be the most profitable. Therefore, while horticultural conclusions for growers with similar growing conditions, such as climate and soils, can be drawn from the study, further economic analysis is necessary prior to providing recommendations.

## 5 Conclusion

In contrast to the general understanding which assumes rootstocks convey the same behavior regardless of scion or that possible interactions observed between cultivar and rootstock diminish overtime, both orchard spacings in the current study observed significant interactions for horticultural parameters of tree survival, trunk size, cumulative yield, and CYE. However, the fruit variables of size, shape, firmness, soluble solids, and color did not have consistent statistical interaction between cultivar and rootstock. While an interaction between stocks has been observed as a product of growing location (Autio et al., 2017; Robinson et al., 2012), the interaction with cultivar is further encouragement for rootstock breeding programs to tailor desired traits for specific economically valuable cultivars. Selection of the best cultivar-scion combinations for growers to have the best horticultural performance is an ongoing work due to changes in consumer preferences or perceptions, increasing material and labor costs, and a changing landscape of disease, pest, and temperatures (Preston et al., 2024). From our results after 17 years, based upon the highest cumulative yields, growers could consider using G.41 for ‘Empire’, and G.11 for ‘Delicious’, ‘Honeycrisp’, and ‘Gala’. However, when also considering plant vigor, G.41 is superior to G.11 as it filled the space and reach the top trellis wire for ‘Empire’, ‘Delicious’, and ‘Honeycrisp’. The modest growth of G.41 and G.11 on ‘Gala’ are both good options. Regarding ‘Mustu’, the highest cumulative yields were similar between G.11, M.9Pajam2, and G.14, yet while growers should avoid using these rootstocks due to excessive vegetative growth and lower yield efficiency values. G.11 and M.9Pajam2 for ‘Mustu’ as the larger yields perhaps do not justify the larger tree size and options including G.41 and G.202 may yield appropriate balance of yield and vigor. Although this work provides several recommendations, our results report interactions observed within Western New York growing conditions and potentially cannot be applied to other growing regions but nonetheless highlight how an interactive effect can influence long-term orchard performance.

## Data Availability

The original contributions presented in the study are included in the article/**Supplementary Material**. Further inquiries can be directed to the corresponding author.
