# Supplementary material for: Rootstock effect on horticultural performance and fruit quality is not uniform across five commercial apple cultivars in western New York
Source: Front Plant Sci. 2025 Mar 10;16:1552625. doi: 10.3389/fpls.2025.1552625 (PMC11930806; doi:10.3389/fpls.2025.1552625)
Supplement: Supplementary file 1 [file Table1.docx]

**Supplemental Table 1.** Least square mean trunk cross sectional area values (TCSA, cm^2^) by cultivar and rootstock after 17 years (2007-2023) of the dwarfing rootstocks in Geneva, NY.

| Rootstock | Empire | Delicious | Honeycrisp | Gala | Mutsu | Mean |
| --- | --- | --- | --- | --- | --- | --- |
| B.9 | 17 c | 13.2 e | 24.2 c | 29.8 f | 56.7 d | 28.2 e |
| G.65 | 15.4 bc | 21.5 de | 23 c | 42.3 ef | 51.5 d | 30.7 e |
| CG.4210 | 30.8 abc | 22.8 cde | 38.3 bc | 55.9 abcdef | 46.7 d | 38.9 de |
| M.9T337 | 38.7 abc | 35.5 bcd | 45 b | 67.3 abcd | 86.4 bcd | 54.6 cd |
| G.202 | 49 ab | 56.6 a | 47 b | 44.4 def | 83.9 cd | 56.2 bcd |
| G.41 | 55.3 a | 49.3 ab | 50.8 ab | 58.3 bcde | 83.4 cd | 59.4 bc |
| G.11 | 29.5 abc | 42.8 abc | 40.6 bc | 54.4 cde | 123.4 abc | 58.1 bcd |
| M.26 | 61.5 a | 50.6 ab | 50.8 ab | 74.2 abc | 146 ab | 76.6 a |
| M.9Pajam2 | 32.3 abc | 54.6 a | 48.3 b | 81.3 ab | 149.2 a | 73.1 ab |
| G.814 | 40.8 abc | 53.4 ab | 66.7 a | 89.7 a | 141.5 ab | 78.4 a |
| Mean | 37.0 c | 40.0 c | 43.5 c | 59.8 b | 96.9 a |  |

^z^ Cultivars and rootstocks listed in order of increasing TCSA. Letters between cultivar and rootstock means are shown following Tukey’s HSD (*P* = 0.05). Letters between rootstocks within each cultivar are shown using t-tests after a Bonferroni adjustment (*P* = 0.001).

**Supplemental Table 2.** Least square mean cumulative yield values (kg tree^-1^) by cultivar and rootstock after 17 years (2007-2023) of the surviving dwarfing rootstocks in Geneva, NY.

| Rootstock | Empire | Delicious | Honeycrisp | Gala | Mutsu | Mean |
| --- | --- | --- | --- | --- | --- | --- |
| B.9 | 150.1 cd | 111.2 c | 174.8 cd | 215.3 c | 299.4 abc | 190.2 d |
| G.65 | 115.5 d | 123.4 c | 114.8 d | 241.8 bc | 237.1 c | 168.7 d |
| CG.4210 | 392.1 ab | 201.5 bc | 277.2 ab | 342.5 abc | 312.4 abc | 302.8 bc |
| M.9T337 | 248.6 bcd | 252.1 ab | 270.3 ab | 382.8 a | 334.8 abc | 298 c |
| G.202 | 358.3 ab | 339.5 a | 269.8 ab | 337 abc | 262.2 bc | 315.5 bc |
| G.41 | 403.6 a | 329.6 ab | 302.1 ab | 424.4 a | 293.2 abc | 352.5 abc |
| G.11 | 387.4 ab | 361.6 a | 326.6 a | 452.2 a | 422.5 ab | 392.4 a |
| M.26 | 286.0 abc | 300.4 ab | 231.8 bc | 358.6 ab | 355.1 abc | 306.5 c |
| M.9Pajam2 | 155.7 bcd | 318.7 ab | 271.3 ab | 445.3 a | 437.2 a | 322.6 bc |
| G.814 | 352.4 ab | 361.5 a | 322.4 a | 425.1 a | 404.9 abc | 370.7 ab |
| Mean | 284.4 b | 274.1 b | 255.5 b | 361.1 a | 335 a |  |

^z^ Cultivars and rootstocks listed in order of increasing TCSA. Letters between cultivar and rootstock means are shown following Tukey’s HSD (*P* = 0.05). Letters between rootstocks within each cultivar are shown using t-tests after a Bonferroni adjustment (*P* = 0.001).

**Supplemental Table 3.** Least square mean cumulative yield efficiency values (CYE, [total kg tree^-1^] / cm^2^ final TCSA) by cultivar and rootstock after 17 years (2007-2023) of the dwarfing rootstocks in Geneva, NY.

| Rootstock | Empire | Delicious | Honeycrisp | Gala | Mutsu | Mean |
| --- | --- | --- | --- | --- | --- | --- |
| B.9 | 8.9 ab | 8.4 a | 7.5 ab | 7.4 ab | 5.3 b | 7.5 bc |
| G.65 | 7.3 bc | 6.0 b | 5.1 b | 5.8 bc | 4.7 bc | 5.8 de |
| CG.4210 | 11.6 a | 8.8 a | 7.3 ab | 6.1 abc | 7.3 a | 8.5 ab |
| M.9T337 | 6.8 bc | 7.3 ab | 6.1 b | 5.7 bc | 4.6 bcd | 6.1 de |
| G.202 | 7.6 b | 6.1 b | 5.9 b | 7.9 a | 3.3 cde | 6.1 de |
| G.41 | 8.0 ab | 7.1 ab | 6.0 b | 7.9 a | 3.7 bcde | 6.5 cd |
| G.11 | 11.8 a | 8.5 a | 9.1 a | 8.6 a | 3.5 cde | 9.7 a |
| M.26 | 4.7 c | 6.2 b | 4.6 b | 4.9 c | 2.7 e | 4.6 f |
| M.9Pajam2 | 4.8 bc | 5.9 b | 5.6 b | 5.4 bc | 3.0 cde | 5.0 ef |
| G.814 | 8.6 ab | 7.2 ab | 5.0 b | 4.9 c | 2.9 de | 5.7 de |
| Mean | 8.8 a | 7.2 b | 6.2 c | 6.5 bc | 4.1 d |  |

^z^ Cultivars and rootstocks listed in order of increasing TCSA. Letters between cultivar and rootstock means are shown following Tukey’s HSD (*P* = 0.05). Letters between rootstocks within each cultivar are shown using t-tests after a Bonferroni adjustment (*P* = 0.001).

**Supplemental Table 4.** Least square mean crop load adjusted fruit size values (g) by cultivar and rootstock after 17 years (2007-2023) of the surviving dwarfing rootstocks in Geneva, NY.

| Rootstock | Empire | Delicious | Honeycrisp | Gala | Mutsu | Mean |
| --- | --- | --- | --- | --- | --- | --- |
| B.9 | 170.6 abc | 201.9 a | 206.1 a | 137.6 b | 287.1 a | 201.6 bc |
| G.65 | 153.1 bc | 211.1 a | 191.3 a | 162.1 a | 310.5 a | 205.6 bc |
| CG.4210 | 157.4 abc | 205.0 a | 223.2 a | 138.0 ab | 255.8 a | 193.3 c |
| M.9T337 | 181 ab | 222.3 a | 239.3 a | 145.9 ab | 276.0 a | 213.0 abc |
| G.202 | 164 abc | 221.0 a | 228.9 a | 133.2 b | 285.8 a | 207.7 bc |
| G.41 | 158 abc | 204.2 a | 234.5 a | 146.7 ab | 289.1 a | 206.5 bc |
| G.11 | 191.1 a | 232.5 a | 237.9 a | 146.6 ab | 327.5 a | 227.6 a |
| M.26 | 165.9 abc | 209.5 a | 209.9 a | 148.0 ab | 314.1 a | 209.2 abc |
| M.9Pajam2 | 147.8 c | 228.1 a | 234.7 a | 155.1 ab | 330.5 a | 218.9 ab |
| G.814 | 152.5 c | 204.3 a | 230.7 a | 144.9 ab | 314.6 a | 209.6 abc |
| Mean | 165.7 c | 213.6 b | 223.3 b | 146.3 d | 297.5 a |  |

^z^ Cultivars and rootstocks listed in order of increasing TCSA. Letters between cultivar and rootstock means are shown following Tukey’s HSD (*P* = 0.05). Letters between rootstocks within each cultivar are shown using t-tests after a Bonferroni adjustment (*P* = 0.001).

**Supplemental Table 5**. Least square mean trunk cross sectional area (TCSA) values (cm^2^) by cultivar and rootstock after 17 years (2007-2023) of the semi-dwarfing rootstocks in Geneva, NY.

| Rootstock | Delicious | Honeycrisp | Empire | Gala | Mustu | Mean |
| --- | --- | --- | --- | --- | --- | --- |
| G.935 | 45.4 c | 54.9 c | 71.6 b | 72.5 c | 132.0 b | 75.9 c |
| G.214 | 55.7 bc | 59.5 c | 48.2 b | 76.1 c | 159.5 b | 79.5 c |
| M.26 | 59.4 bc | 62.6 bc | 65.1 b | 101.8 bc | 170.1 b | 91.5 c |
| G.30 | 75.0 abc | 75.3 bc | 68.0 b | 103.1 bc | 214.3 ab | 107.0 c |
| G.222 | 77.7 ab | 78.7 bc | 57.0 b | 106.3 bc | 198.1 b | 104.0 c |
| M.7 | 95.3 a | 99.3 b | 134.4 a | 148.1 ab | 238.0 ab | 143.5 b |
| B.118 | 99.0 a | 146.5 a | 169.1 a | 188.1 a | 322.1 a | 185.3 a |
| Mean | 72.8 d | 81.9 cd | 88 c | 113.7 b | 205.5 a |  |

^z^ Cultivars and rootstocks listed in order of increasing TCSA. Letters between cultivar and rootstock means are shown following Tukey’s HSD (*P* = 0.05). Letters between rootstocks within each cultivar are shown using t-tests after a Bonferroni adjustment (*P* = 0.002).

**Supplemental Table 6.** Least square mean cumulative yield (kg tree^-1^) by cultivar and rootstock after 17 years (2007-2023) of the dwarfing rootstocks in Geneva, NY.

| Rootstock | Delicious | Honeycrisp | Empire | Gala | Mustu | Mean |
| --- | --- | --- | --- | --- | --- | --- |
| G.935 | 356.2 ab | 348.4 bc | 497.4 a | 503.2 a | 563.1 a | 454.5 abc |
| G.214 | 354.1 ab | 341.2 bc | 373.7 a | 561.5 a | 553 a | 440.6 bc |
| M.26 | 302.9 b | 286.9 c | 383.1 a | 577.3 a | 504.2 a | 409.5 c |
| G.30 | 478.5 a | 424.2 ab | 474.7 a | 647.9 a | 580.9 a | 520.4 a |
| G.222 | 397.5 ab | 388.9 bc | 403.5 a | 525 a | 471.4 a | 437.3 bc |
| M.7 | 327 b | 349.8 bc | 419.1 a | 459.8 a | 435.8 a | 396.6 c |
| B.118 | 413.2 ab | 534.6 a | 511.2 a | 594.7 a | 509.8 a | 511.9 ab |
| Mean | 375.4 c | 381.6 c | 437.8 b | 554.2 a | 515.9 a |  |

^z^ Cultivars and rootstocks listed in order of increasing TCSA. Letters between cultivar and rootstock means are shown following Tukey’s HSD (*P* = 0.05). Letters between rootstocks within each cultivar are shown using t-tests after a Bonferroni adjustment (*P* = 0.002).

**Supplemental Table 7.** Least squared mean cumulative yield efficiency values ([total kg tree^-1^] / cm^2^ final TCSA) by cultivar and rootstock after 17 years (2007-2023) of the semi-dwarfing rootstocks in Geneva, NY.

| Rootstock | Delicious | Honeycrisp | Empire | Gala | Mustu | Mean |
| --- | --- | --- | --- | --- | --- | --- |
| G.935 | 7.8 a | 6.4 a | 9.5 a | 6.7 a | 4.2 a | 6.8 a |
| G.214 | 6.8 ab | 6 ab | 7.8 a | 7.7 a | 3.5 ab | 6.4 ab |
| M.26 | 5.7 abc | 4.8 bcd | 6 ab | 5.9 a | 3.2 bc | 5.1 c |
| G.30 | 6.6 ab | 5.6 ab | 7.3 a | 6.4 a | 2.7 bcd | 5.7 bc |
| G.222 | 5.7 abc | 5.2 abc | 7.5 a | 5.2 ab | 2.5 cd | 5.2 c |
| M.7 | 3.4 c | 3.9 cd | 3.2 b | 3.2 b | 2.1 de | 3.2 d |
| B.118 | 4.3 bc | 3.7 d | 3.1 b | 3.3 b | 1.6 e | 3.2 d |
| Mean | 5.8 ab | 5.1 c | 6.3 a | 5.5 bc | 2.8 d |  |

^z^ Cultivars and rootstocks listed in order of increasing TCSA. Letters between cultivar and rootstock means are shown following Tukey’s HSD (*P* = 0.05). Letters between rootstocks within each cultivar are shown using t-tests after a Bonferroni adjustment (*P* = 0.002).

**Supplemental Table 8.** Least squared mean crop load adjusted fruit size values (g) by cultivar and rootstock after 17 years (2007-2023) of the semi-dwarfing rootstocks in Geneva, NY.

| Rootstock | Delicious | Honeycrisp | Empire | Gala | Mutsu | Mean |
| --- | --- | --- | --- | --- | --- | --- |
| G.935 | 221.5 | 241.0 | 159.8 | 141.5 | 321.0 | 215.8 |
| G.214 | 188.0 | 242.4 | 151.3 | 142.9 | 322.4 | 209.5 |
| M.26 | 208.4 | 233.3 | 168.7 | 154.1 | 317.6 | 215.8 |
| G.30 | 208.0 | 236.3 | 163.4 | 142.1 | 338.0 | 218.8 |
| G.222 | 215.9 | 238.7 | 166.2 | 148.1 | 305.8 | 215.8 |
| M.7 | 205.5 | 240.9 | 161.8 | 151.3 | 323.5 | 215.5 |
| B.118 | 210.1 | 242.9 | 164.2 | 151.9 | 325.4 | 219.5 |
| Mean | 208.7 c | 236.4 b | 167.4 d | 148.7 e | 317.9 a |  |

^z^ Cultivars and rootstocks listed in order of increasing TCSA. Letters between cultivar and rootstock means are shown following Tukey’s HSD (*P* = 0.05). Letters between rootstocks within each cultivar are shown using t-tests after a Bonferroni adjustment (*P* = 0.002).
